# The effect of gut microbiota dysbiosis on patients with preeclampsia

**DOI:** 10.3389/fcimb.2022.1022857

**Published:** 2023-01-04

**Authors:** Yefang Zhao, Bingjie Wang, Xiaoling Zhao, Dan Cui, Shaoke Hou, Hongzhen Zhang

**Affiliations:** ^1^ Department of Obstetrics and Gynecology, First Hospital of Hebei Medical University, Shijiazhuang, Hebei, China; ^2^ Department of Obstetrics, Xingtai People’s Hospital, Affiliated Hospital of Hebei Medical University, Xingtai, Hebei, China

**Keywords:** preeclampsia, gut microbiota, microbial α diversity, microbial β diversity, proinflammatory factor

## Abstract

**Purpose:**

To compare the difference of gut microbiota between preeclampsia (PE) and healthy normal pregnant women, providing new therapeutic strategy for preeclampsia.

**Methods:**

Forty-one PE patients and 45 age- and pre-pregnancy body mass index- matched healthy controls were enrolled from Nov 2021 to May 2022 in this retrospective case-control study. Fecal microbiota was detected by 16S rRNA gene sequencing, followed by bioinformatics analysis including microbial α diversity, microbial β diversity, and linear discriminant analysis effect size (LEfSe) analysis. Serum inflammatory factors were also detected and compared between the two groups.

**Results:**

There were significant differences in Bacteroidetes (2.68% in PE patients vs 11.04% in healthy controls, *P <* 0.001), Proteobacteria (4.04% in PE patients vs 1.22% in healthy controls, *P* = 0.041), and Fusobacteria (1.07% in PE patients vs 0.01% in healthy controls, *P* = 0.042) between the two groups at the phylum level. Microbial α diversity was lower in PE patients than that in healthy controls. In addition, there was significant difference in microbial β diversity between the two groups. LEfSe analysis showed that there are 24 different taxa between the two groups. The levels of proinflammatory factors including serum tumor necrosis factor-α and Interleukin-6 were statistically significant higher in PE patients than those in healthy controls (both *P* < 0.001), while there were no significant differences in the levels of serum anti-inflammatory factors including Interleukin-4 and Interleukin-10 between the two groups (*P* = 0.234 and *P* = 0.096, respectively).

**Conclusion:**

PE patients demonstrated gut microbiota disturbances and increasing serum proinflammatory factors, leading to a better understanding of the relationship between the gut microbiota dysbiosis and PE.

## Introduction

Preeclampsia (PE) manifests new-onset hypertension associated with pregnancy, usually occurring after 20 weeks of gestation and most often at term (Gestational hypertension and preeclampsia: ACOG practice bulletin summary, number 222, 2020). PE complicates about 3–5% of all pregnancies and it is estimated to cause at least 42 000 maternal deaths annually (Abalos et al., 2013; Ananth et al., 2013; Say et al., 2014). It is often accompanied by one of the following conditions: renal failure, liver dysfunction, hematological or neurological abnormalities, intrauterine growth restriction, and uteroplacental insufficiency (Mol et al., 2016). PE can present in many ways; It can be diagnosed after a woman presents with a seizure, breathlessness, severe epigastric pain, and massive placental abruption, or diagnosed at a routine antenatal consultation if a woman is asymptomatic but hypertensive (Chappell et al., 2021). Nowadays, the pathogenesis of PE remains unclear and the effective treatment for PE is delivery of the placenta to stop the progression of the disease. Therefore, PE is one of the most important causes of iatrogenic preterm birth and lacks reliable prediction methods, leading to serious and even fatal complications for both mother and fetus (Hampton, 2020). It is thus of particular interest to find effective and accessible clinical biomarkers of PE.

Gut microbiota is a complex and huge community of microorganism species living in the digestive tracts, including bacteria, fungi, viruses, archaea and protozoa. It is capable of producing a diverse range of compounds that regulate the activity of distal organs (Viennois and Chassaing, 2018). The ecosystem contributes to a large amount of physiological functions: fermentation of indigestible dietary components and vitamin synthesis, defenses against pathogens, host immune system maturation and maintenance of gut barrier function (Alam and Neish, 2018; Cheng et al., 2019).Any alteration in the structure and composition of gut microbiota can be linked to host metabolic abnormalities and systemic inflammation, may involving in many diseases including obesity, type 2 diabetes, prediabetes, cardio-metabolic diseases, non-alcoholic liver disease, and malnutrition (Li et al., 2017; Alam and Neish, 2018; Sun et al., 2018; Sircana et al., 2018; Cheng et al., 2019; Safari and Gérard, 2019; Fan and Pedersen, 2021). Fecal transplantation from hypertensive human donors to germ-free mice elevated blood pressure, confirming the effect of gut microbiota dybiosis (Safari and Gérard, 2019). The mechanism of intestinal flora regulation leading to chronic hypertension may be through its metabolites: short chain fatty acids (Armani et al., 2017) (SCFAs, including butyric acid, propionic acid and acetic acid), oxidation of trimethylamine N-oxide (TMAO), impairment of intestinal mucosal barrier function, reduction of beneficial bacteria synthesis, and production of a variety of inflammatory factors to increase blood pressure (Natarajan et al., 2016).

PE is characterized by hypertension and multiple organ dysfunction after 20 weeks of gestation with normal blood pressure in the first trimester. Previous studies consider that the originating cause of PE is microbial infection. Bacterial products such as Lipopolysaccharides (LPS), which is highly inflammatory and could stimulate an innate immune response that aggravates inflammation action further (Armani et al., 2017). Disruption of intestinal barrier caused by gut microbiota dysbiosis can increase the level of LPS, enhancing local and systemic inflammation (Natarajan et al., 2016; Kell and Kenny, 2016; Robles-Vera et al., 2018). Attenuation of proinflammatory state led to enhanced intestinal barrier function and thus had therapeutic effect in mouse models for high fat diet (HFD)-induced obesity, providing guidance for treating PE (Luck et al., 2015; Natarajan et al., 2016). However, only a few studies have illustrated the relationship among gut microbiota dysbiosis, inflammation and PE (Garidou et al., 2015). The purpose of this study is to investigate the relationship among gut microbiota among the relationship between gut microbiota dysbiosis, inflammatory factors and PE, uncovering new light for PE prediction and precise intervention.

## Materials and methods

### Study design

The study is designed as a retrospective case-control study. The study followed the tenets of the Declaration of Helsinki and was approved by the ethics review board of First Hospital of Hebei Medical University (approval number, 20220215). All subjects provided written informed consent.

### Study subjects

A total of 86 cases of singleton pregnancy mother at the Department of Obstetrics and Gynecology of First Hospital of Hebei Medical University and Department of Obstetrics of Xingtai People’s Hospital from Nov 2021 to May 2022 were included. Forty-one subjects with PE patients were enrolled into the PE group and 45 age- and pre-pregnancy body mass index (BMI)-matched healthy controls were recruited into the normal group.

According to the present American College of Obstetrics and Gynecologists criteria (Gestational hypertension and preeclampsia: ACOG practice bulletin summary, number 222, 2020), PE was defined as a multisystemic disorder characterized by new onset of hypertension (systolic blood pressure (SBP) ≥ 140 mmHg and/or diastolic blood pressure (DBP) ≥ 90 mmHg) and proteinuria (> 300 mg/24h) arising after 20 weeks of gestation in a previously normotensive woman. It is also characterized by new-onset hypertension with new-onset of any conditions including thrombocytopenia (platelet count ≤ 100×10^9^/L), renal insufficiency (serum creatinine concentrations ≥ 1.1 mg/dL or a doubling of the serum creatinine concentration in the absence of other renal disease), impaired liver function (elevated blood concentrations of liver transaminases to twice normal concentration), and pulmonary edema or new-onset headache unresponsive to medication.

The inclusion criteria were as follows: 1) single pregnancy, 2) 20-41 weeks of gestation, 3) well communication skills, 4) no mental disease, 5) agreed to participate in the study and signed informed consent. The exclusion criteria were as follows: 1) twin or multiple pregnancies, 2) with diabetes, chronic hypertension, renal diseases, malignancies, inflammatory bowel disease or other pregnancy complications, 3) history of smoking, alcohol, and narcotic drug use, 4) recent acute or chronic infection and use of antibiotics, pharmaceutical grade probiotics or immunosuppressive therapy within 1 month, 5) age < 18 years old, 6) history of gastrointestinal surgery, 7) mental abnormalities or mental retardation.

### Sample collection and 16S rRNA sequencing

The fecal samples were collected into sterile tube with a screw cap attached scoop by the subjects and were frozen at -20 to -40°C. being transferred to the laboratory on dry ice within 24 h of collection and stored at −80°C until DNA extraction.

### DNA extraction

Total genomic DNA samples were extracted using the OMEGA Soil DNA Kit (M5635-02) (Omega Bio-Tek, Norcross, GA, USA) following the manufacturer’s instructions. The quantity and quality of extracted DNAs were measured using a NanoDrop NC2000 spectrophotometer (Thermo Fisher Scientifific, Waltham, MA, USA) and agarose gel electrophoresis, respectively.

### 16S rRNA gene amplicon sequencing

Polymerase chain reaction (PCR)amplification of the bacterial 16S rRNA genes V3–V4 region was performed using the forward primer 338F (5’-ACTCCTACGGGAGGCAGCA-3’) and the reverse primer 806R (5’-GGACTACHVGGGTWTCTAAT-3’) (Ma et al., 2004; Monteiro-Sepulveda et al., 2015). Sample-specific 7-bp barcodes were incorporated into the primers for multiplex sequencing. The PCR components contained 5 μl of buffer (5×), 0.25 μl of Fast pfu DNA Polymerase (5U/μl), 2 μl (2.5 mM) of dNTPs, 1 μl (10 uM) of each Forward and Reverse primer, 1 μl of DNA Template, and 14.75 μl of ddH_2_O. Thermal cycling consisted of initial denaturation at 98°C for 5 min, followed by 25 cycles of denaturation at 98°Cfor 30 s, annealing at 53°C for 30 s, extension at 72°C for 45 s, and a final extension of 5 min at 72°C. PCR amplicons were purified with Vazyme VAHTSTM DNA Clean Beads (Vazyme, Nanjing, China) and quantified using the Quant-iT PicoGreen dsDNA Assay Kit (Invitrogen, Carlsbad, CA, USA). After the individual quantification step, amplicons were pooled in equal amounts and pair-end 2×250 bp sequencing was performed using the Illlumina NovaSeq platform with NovaSeq 6000 SP Reagent Kit (500 cycles) at Shanghai Personal Biotechnology Co., Ltd (Shanghai, China).

### Sequence Analysis

Microbiome bioinformatics were performed with QIIME2 2019.4 with slight modification according to the official tutorials (https://docs.qiime2.org/2019.4/tutorials/). Briefly, raw sequence data were demultiplexed using the demux plugin following by primers cutting with cutadapt plugin. Sequences were then quality-filtered, denoised, merged and chimera removed using the DADA2 plugin. Non-singleton amplicon sequence variants (ASVs) were aligned with MAFFT and used to construct a phylogeny with FastTree2. Alpha-diversity metrics (Chao1, Observed species, Shannon, Simpson, Faith’s PD, Pielou’s evenness, and Good’s coverage) beta diversity metrics (weighted UniFrac, unweighted UniFrac, Jaccard distance, and Bray-Curtis dissimilarity) were estimated using the diversity plugin with samples being rarefied to 28390 sequences per sample. Taxonomy was assigned to ASVs using the classify-sklearn naive Bayes classifier in feature-classifier plugin against the gg_13 Database.

### Bioinformatics and statistical analysis

Sequence data analyses were mainly performed using QIIME2 and R packages (v3.2.0). ASV-level alpha diversity indices including Chao1 richness estimator, observed species, Shannon diversity index, Simpson index, Faith’s PD, Pielou’s evenness, and Good’s coverage were calculated using the ASV table in QIIME2 and were visualized as box plots. ASV-level ranked abundance curves were generated to compare the richness and evenness of ASVs among samples. Beta diversity analysis was performed to investigate the structural variation of microbial communities across samples using UniFrac distance metrics and were visualized *via* principal coordinate analysis (PCoA). Principal component analysis (PCA) was also conducted based on the genus-level compositional profiles. The significance of differentiation of microbiota structure among groups was assessed by ANOSIM (Analysis of similarities) using QIIME2. LEfSe was performed to detect different abundant taxa across groups using the default parameters.

### Quantification of serum inflammatory factors concentrations

Serum chemokine assay was performed by enzyme-linked immunosorbent assay (ELISA). The expressions of serum interleukin-6 (IL-6), tumor necrosis factor-α (TNF-α), interleukin-4 (IL-4), and interleukin-10 (IL-10) were measured by Abcam ELISA Kit. The minimum detection limits were 7.8 pg mL^-1^ for IL-6, 15.6 pg mL^-1^ for TNF-α, 15.6 pg mL^-1^ for IL-4, and 15.6 pg mL^-1^ for IL-10. The maximum detection limits were 500 pg mL^-1^ for IL-6, 500 pg mL^-1^ for TNF-α, 1000 pg mL^-1^ for IL-4, and 1000 pg mL^-1^ for IL-10. These kits are purchased from Proteintech.

### Statistical analysis

Statistical Product and Service Solutions (SPSS) software (version 24.0; SPSS, Inc., IL, USA) and GraphPad Prism software (version 7.0; GraphPad Software, Inc., San Diego, CA) were used for data analysis and figures production. The Shapiro-Wilk test was used to judge the normal distribution of data. Normal distribution data was described as mean ± standard deviation (SD and skewed distribution date was described as median (25th-75^th^ quartiles). Statistical analysis between groups were analyzed using the Wilcoxon’s rank-sum test, Student’s t-test or one-way analysis of variance. Rate comparisons were performed with Pearson’s χ2 test or Fisher’s exact test. All tests were two sided, and *P* < 0.05 was considered as statistically significant.

## Results

### Baseline characteristics

No significant differences were observed in maternal age, gravidity, parity, pre-pregnancy BMI between the two groups (all *P* > 0.05). In addition, there were no significant differences in pregnancy outcomes including placental abruption, postpartum hemorrhage, fetal growth restriction, perinatal death, fetal distress, cardiac insufficiency, and oligohydramnios between the two groups (all *P* > 0.05). No significant differences were observed in white blood count, percentage of neutrophils and lymphocytes between the two groups (all *P* > 0.05). The PE group had lower weight gain during pregnancy, birth weight, gestational weeks on delivery compared to the normal group (all *P* < 0.05), while proportion of cesarean section and induced preterm birth < 34W and SBP and DBP were higher in the PE group (all *P* < 0.05, **Table 1**).

**Table 1:** Baseline characteristics of the PE and normal groups.

	PE (n = 41)	No (n = 45)	*P-*value
Maternal age (years old)	31.9 ± 4.2	31.0 ± 4.6	0.385
Gravidity (N)	3 (2, 3)	2 (1, 3)	0.073
Parity (N)	1(0, 1)	1(0, 1)	0.083
Pre-pregnancy BMI (Kg/m^2^)	24.67 ± 4.64	22.91 ± 4.00	0.064
Gain weight (Kg)	12.67 ± 5.97	15.18 ± 4.16	0.025
BP at fecal collection (mmHg)			
Systolic	152.41 ± 19.24	116.24 ± 10.80	< 0.001
Diastolic	100.22 ± 9.92	76.38 ± 7.44	< 0.001
24-h proteinuria collection (g)	1.05 (0.21, 2.40)	Not available	
Birth weight (g)	2582.93 ± 781.70	3425.56 ± 453.81	< 0.001
Gestational weeks on delivery (weeks)	36.21 ± 3.03	39.19 ± 1.12	< 0.001
Pregnancy outcome [number (%)]			
Placental abruption	3 (7.32)	0 (0.00)	0.104
Postpartum hemorrhage	2 (4.88)	2 (4.44)	1.000
Induced preterm birth<34weeks	12 (29.27)	1 (2.22)	< 0.001
Cesarean section	32 (78.05)	23 (51.11)	0.009
Fetal growth restriction	2 (4.88)	1 (2.22)	0.063
Perinatal death	0 (0.00)	1 (2.22)	1.000
Fetal Distress	2 (4.88)	1 (2.22)	0.063
Cardiac insufficiency	1 (2.44)	0 (0.00)	0.477
Oligohydramnios	1 (2.44)	0 (0.00)	0.477
White blood cell count at fecal collection(10^9^/L)	7.83 ± 1.85	7.84 ± 1.76	0.981
Percentage of neutrophils at fecal collection (%)	72.88 ± 657	72.28 ± 7.05	0.685
Percentage of lymphocytes at fecal collection (%)	20.78 ± 6.11	21.13 ± 6.49	0.797

Normal distribution data was described as mean ± standard deviation (SD and skewed distribution date was described as median (25th-75^th^ quartiles). PE: preeclampsia. P ≤ 0.05 was considered of significant difference.

### Varying composition of gut microbiota in the PE group and the healthy control group

**Figure 1:**
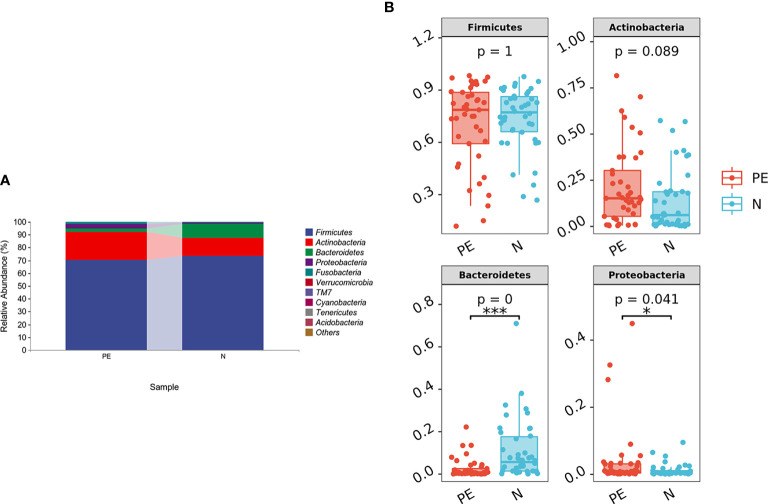
The distribution of gut microbiota at the phylum level in the different groups. The top 10 bacteria **(A)** and top 4 bacteria **(B)** at the phylum level of each group were showed. PE, preeclampsia; N, the normal group. *P < 0.05, ***P < 0.001.

In this study, 86 fecal samples were collected for sequencing. At the phylum level, the relative abundance of the top 10 taxa showed that the dominate phylum bacteria were Firmicutes, Actinobacteria, Bacteroidetes, and Proteobacteria (**Figure 1A**).

The relative abundance of Bacteroidetes was significantly lower in the PE group compared with the normal group (*P* < 0.001) and the relative abundance of Proteobacteria was higher in the PE group (*P* = 0.041). However, there were no significant differences in Firmicutes and Actinobacteria between the two groups (both *P* > 0.05, **Figure 1B**). Varying Alpha and Beta Diversity in the Two Groups

**Figure 2:**
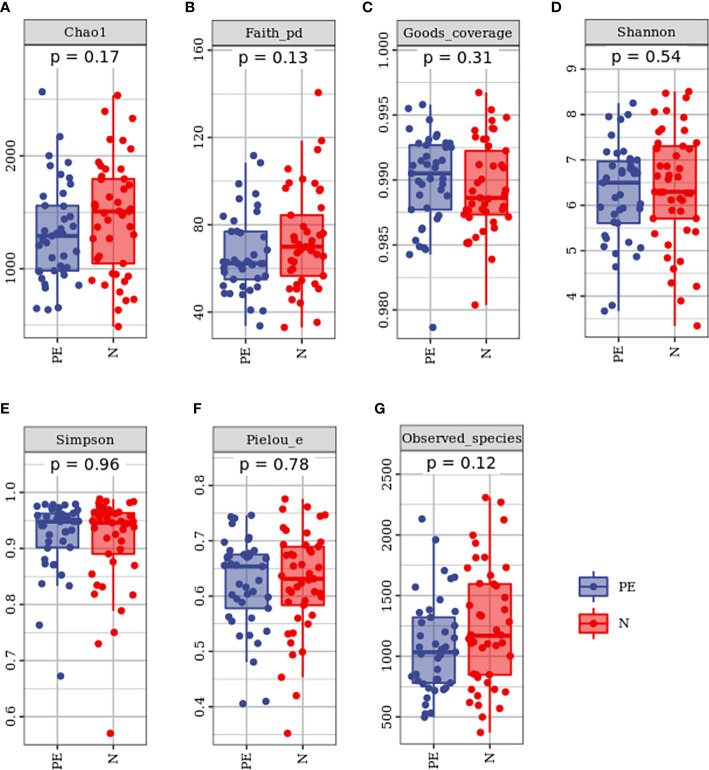
Varying alpha diversity of gut microbiota in the two groups. Chao1 diversity index **(A)**, Faith-PD **(B)**, Goods coverage **(C)**, Shannon diversity index**(D)**, Simpson diversity index **(E)**, Pielou-e **(F)**, and Observed species **(G)** in the PE group were lower than those in the normal group with no significant differences. PE, preeclampsia; N, the normal group.

Compared to the normal group, microbial alpha diversity was slight lower in the PE group, based on the Chao 1 diversity index (*P* = 0.17), Shannon diversity index (*P* = 0.54), Simpson diversity index (*P* = 0.54), Observed species diversity index (*P* = 0.12), Faith’s PD (*P* = 0.13), Good’s coverage (*P* = 0.31) and Pielou e (*P* = 0.78), but with no statistically significant differences (**Figure 2**).

**Figure 3:**
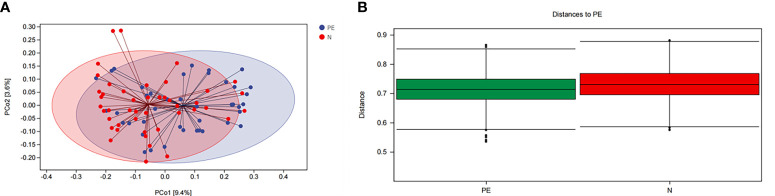
The bacterial microflora composition in beta diversity between the two groups. **(A)** PCoA plot showing the dispersal of microbiota, **(B)** Analysis of similaritied (ANOISM) analysis. PE, preeclampsia; N, the healthy normal group. PCo1, The first principal coordinate; PCo2, The second principal coordinate; PCoA, Principal coordinate analysis.

Microbial beta diversity was evaluated with PCoA plot and ANOISM analysis based on unweighted Unifrac distance analysis. As shown in **Figure 3**. This study showed that the red and the blue points displayed an obvious clustering way. They were on behalf of a separation between the samples from the PE group and the healthy controls based on the PC1 and PC2 scores that accounted for 9.4% and 3.6% of total variations, respectively (**Figure 3A**).

ANOISM analysis indicated that the bacterial microflora composition of the PE group and the healthy singleton group was significantly different (*P* = 0.001, **Figure 3B**).

### Bacteria Taxa differences between the PE group and the normal pregnancy

**Figure 4:**
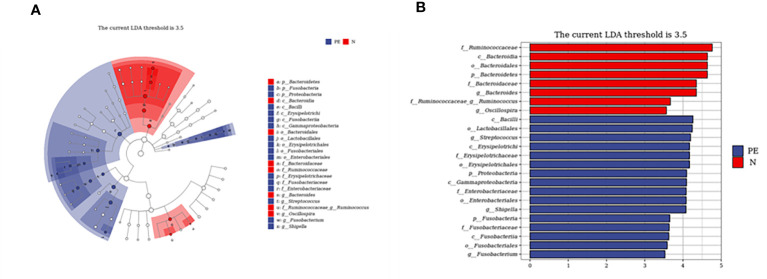
Taxonomic biomarkers of pregnant women between the two groups. Cladogram **(A)** and scores **(B)** of taxonomic biomarkers identified by LDA using LEfSe between the two groups. Color indicates the group in which a differentially abundant taxon is enriched. The LDA scores (log10) > 3.5. PE, preeclampsia; N, the healthy normal group; LEfSe, Linear discriminant analysis combined effect size measurements; LDA, Linear discriminant analysis; P, Phylum; C, Class; O, Order; F, Family; G, Genus.

To further investigate which taxa can serve as the biomarker and have the ability to distinguish the PE patients from the healthy controls, we used the LEfSe analysis to explore the different changes and relative richness of the gut microbiota. A total of 24 abundant taxa were different in the PE group and the normal healthy group, which had a log LDA score>3.5. The relative abundances of the phylum of Bacteroidetes, the class of Bacteroidia, the order of Bacteroidales, the family of Bacteroidaceae and Ruminococcaceae, and the genus of Bacteroides, Ruminococcus and Oscillospira were lower in the PE group than those in the normal group, while the relative abundances of the phylum of Proteobacteria and Fusobacteria, the class of Erysipelotrichi, Bacilli, Fusobacteria, and Gammaproteobacteria, the order of Erysipelotrichales, Enterobacteriales, Fusobacteriales, and Lactobacillales, the family of Erysipelotrichaceae, Enterobacteriaceae, and Fusobacteriaceae, and the genus of Shigella, Fusobacterium, and Streptococcus were higher in the PE group than those in the normal healthy group. The consequences are presented with red and blue colors, showing decrease and increase of abundance in PE group, respectively (**Figures 4A, B**).

### The ratio of Firmicutes and Bacteroidetes (F/B) at the phylum level between the two groups

**Figure 5:**
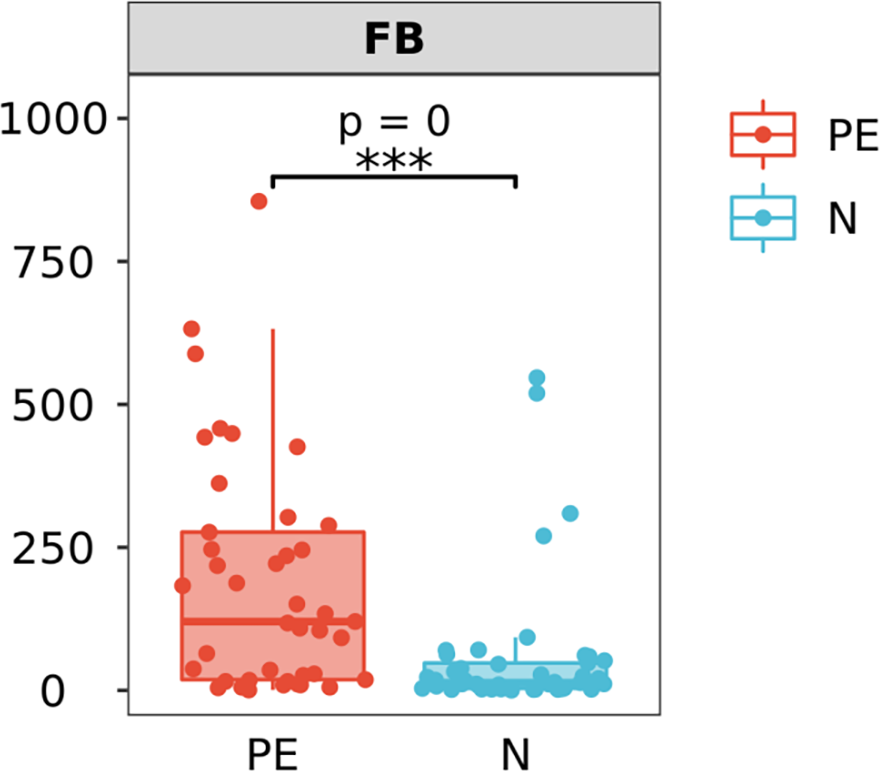
Varying ratios of Firmicutes and Bacteroidetes (F/B) at the phylum level between the two groups. F/B at the phylum level in the PE group is significantly higher than that in the normal group. PE, preeclampsia; N, the healthy normal group. ***P < 0.001.

The F/B level in the PE patients was 120.4 (18.2, 282.5), which was significantly higher than that of the control group [13.5 (3.6, 50.0), *P* < 0.001, **Figure 5**].

### Varying serum inflammatory factors in the two groups

**Figure 6:**
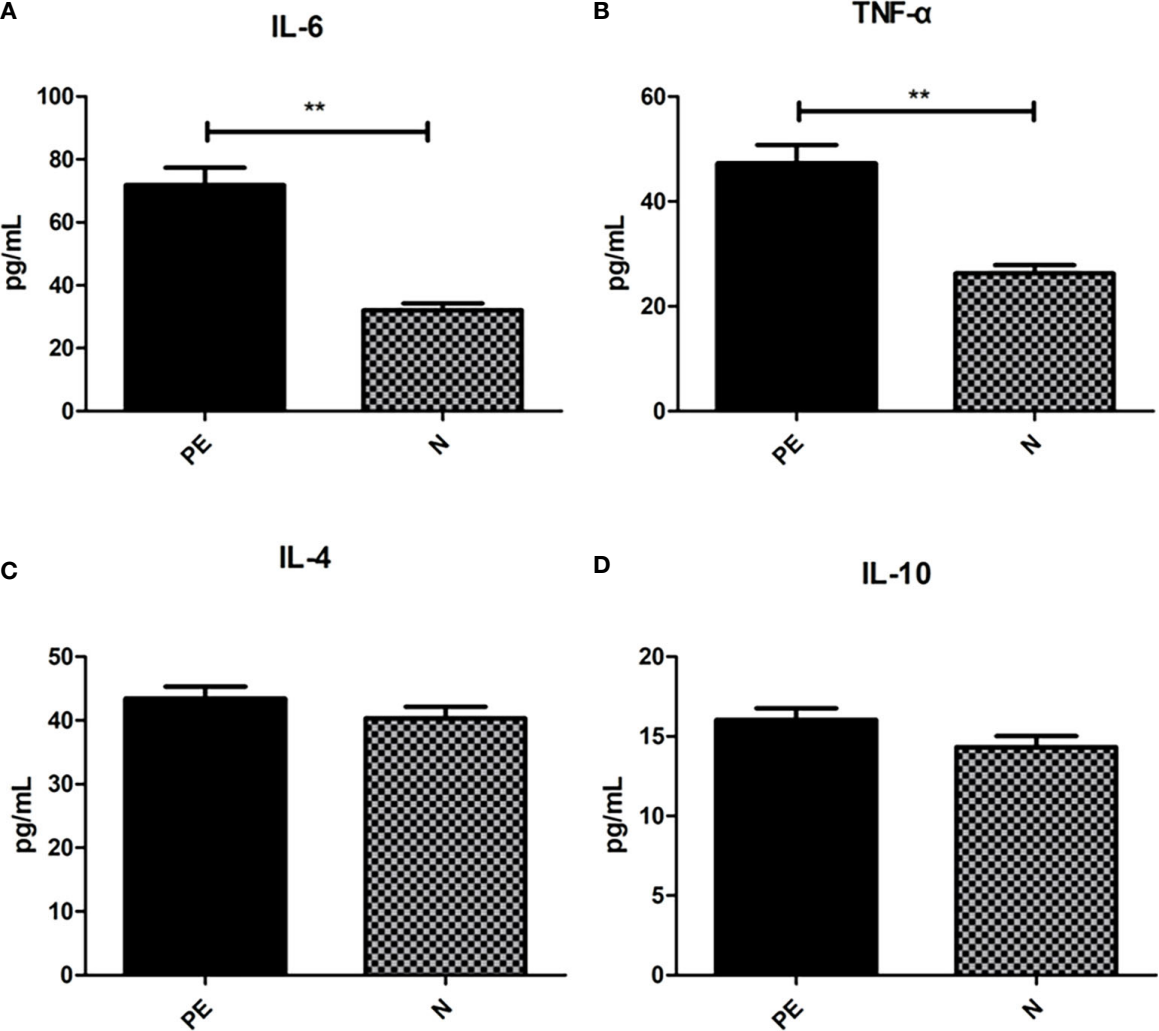
Varying inflammatory factors in the two groups. There were significant differences in the IL-6 **(A)** and TNF-α **(B)** between the two groups. However, no significant differences were observed in IL-6 **(C)** and IL-10 **(D)** between the two groups. PE, preeclampsia; N, the healthy normal group. **P < 0.01.

Shown in **Figure 6**, the serum pro-inflammatory factor IL-6 in the PE patients (71.89 ± 34.85) was higher than that in the normal group (32.04 ± 14.42) with significant difference (*P <*0.001) and TNF-α in the PE patients (47.29 ± 22.41) was significantly higher than that in the normal group (26.26 ± 10.71, *P* < 0.001). However, there were no significant differences in the serum anti-inflammatory factor including IL-4 (43.44 ± 11.85 vs 40.30 ± 12.69, *P* = 0.234) and IL-10 (16.04 ± 4.70 vs 14.32 ± 4.78, *P* = 0.096).

## Discussion

This study showed that there were significant differences in Bacteroidetes, Proteobacteria, and Fusobacteria between the two groups at the phylum level. Microbial alpha diversity was lower in preeclampsia patients than that in healthy controls, but with no statistically significant differences. In addition, there was significant difference in microbial beta diversity between the two groups. LEfSe analysis showed that there are 24 different taxa between the two groups. The levels of proinflammatory factors were statistically significant higher in PE patients than those in healthy controls, while there were no significant differences in the levels of serum anti-inflammatory factors.

Our body is a “super organism” composed of cells and all symbiotic microorganisms. It is a very complex ecosystem (Lynch and Pedersen, 2016). The number of bacteria in adults (3.8×10^13^) is about 1.3 times that of cells (3.0×10^13^) (Sender et al., 2016a), which carries 150 times more genes than our own genome (Ursell et al., 2014). Together with human genes, they affect human immune, nutrition and metabolic processes. There are a certain number of bacterial colonies in the intestinal tract, skin, respiratory tract, reproductive tract and other parts of the human body in contact with the environment. However, the intestinal tract is the most complex colonization environment with the largest number of bacteria in the human body. It is estimated that about 150g microorganisms colonize here, forming the intestinal flora of the human body. Intestinal microflora is composed of a large number of microbial populations with different species. They are established from birth and influenced by mode of birth, infant feeding, lifestyle, medication and the genetics of the host, interact with each other, and constantly exchange information with host cells. They play a vital role in digestion, maintenance and enhancement of intestinal barrier, immune defense, nervous system regulation, nutrition and metabolism, etc (Sender et al., 2016b).

Aberrant gut microbiota lead to the pathogenesis of various common metabolic disorders including obesity, type 2 diabetes, gestational mellitus, non-alcoholic liver disease, cardio-metabolic diseases malnutrition (Fan and Pedersen, 2021) and glomerulonephritis (Ardalan et al., 2022). Recently, some studies have confirmed the causal relationship between intestinal microbial composition and dysfunction and diseases. When diseased faeces are transplanted into sterile mice, the transplanted mice show similar symptoms to the disease, which once again confirms the role of intestinal flora in the occurrence and development of diseases (Ridaura et al., 2013; Li et al., 2017). An vitro and vivo study on the transplantation of PE faeces microbiota confirmed that intestinal beneficial bacteria: Akkermansia muciniphila, propionate or butyrate significantly alleviated the symptoms of preeclampsia rats by promoting autophagy and M2 polarization of macrophages in placental bed, thereby inhibiting inflammation and improving spiral artery recasting (Jin et al., 2022).

Multiple and sometimes overlapping pathologic processes activate a common pathway consisting of endothelial cell activation, intravascular inflammation, and syncytiotrophoblast stress in PE patients. However, the specific etiology of PE remains unclear (Jin et al., 2022). Several mechanisms of disease have been proposed in PE including systemic inflammation, oxidative stress, and vascular endothelial dysfunction, all of which are features of metabolic syndrome (Bakker et al., 2009; Jung et al., 2022).

Animal studies demonstrated that use of antioxidants such as epigallocatechin gallate and resveratrol benefited for the efficacy of nifedipine, the time needed to return to normal blood pressure values and the number of doses needed. They found that antioxidants significantly improved the hypertension and proteinuria and its level could reach normal level. In addition, levels of Toll Like Receptor 4 (TLR4) and inflammatory factors, such as Nuclear Factor κB (NF-κB), IL-6 and Monocyte Chemoattractant Protein-1 (MCP-1), were also significantly decreased compared to the untreated PE group. However the weight of the offspring did not differ from the control group. While, a similar experiment demonstrated that antioxidants also significantly decreased Lipopolysaccharides(LPS)-generated inflammation through upregulation of phosphorylated Protein kinase B (Akt) and significantly increased the weight and number of fetuses to control levels (Funk et al., 2012; Stekkinger et al., 2013). While another study showed that antioxidants together with nifedipine in women with severe PE showed a significantly faster blood pressure decrease than with nifedipine alone and it also increased the interval before a new hypertensive crisis (Spradley, 2017).

Despite few human studies with antioxidants in PE, its effect has been extensively studied in murine models. The use of these antioxidants as a single therapy is still far away, recent studies create a precedent for the use of antioxidants as a coadjuvant against pregnancy complications.

The recent studies showed that gut microbiota play an important role in host nutrition, energy absorption, and immune response to potential pathogens (Gong et al., 2016; Zhou et al., 2017; Shi et al., 2018). Aberrant gut microbiota may contribute to the pathogenesis of various common metabolic diseases (Monteiro-Sepulveda et al., 2015). In human gut microbiota, there are nine phyla including Firmicutes, Bacteroidetes, Actinobacteria, Proteobacteria, etc. Firmicutes is the most abundant phylum, followed by Bacteroidetes, together accounting for 95% of all bacteria (Bäckhed et al., 2004).

Researches demonstrated different results about Microbial alpha diversity between preeclampsia and normal group. One study found that there were no differences between the two groups (Ley et al., 2006), while another study found that microbial alpha diversity was lower in the PE group (Turnbaugh et al., 2006). There was significant difference in microbial beta diversity between the two groups, this was similar to most of the studies (Jia et al., 2008; Funk et al., 2012).

Firmicutes and Actinobacteria were the two most abundant bacteria in pregnant women in this study, while Liu’s study showed that Bacteroidetes was the most abundant bacteria in pregnant women in South China (Hooper et al., 2012). The difference may be due to the different regions of patients. In order to determine whether Bacteroidetes is involved in the pathogenesis of preeclampsia, it is necessary to increase the sample size, enroll patients in different regions, and conduct prospective studies. However, the top ten bacteria at the phylum in our study was similar to the result of Koren’s research (Ley et al., 2006).

Koren et al. demonstrated that gut microbiota changed dramatically from first to third trimester with overall increase in Proteobacteria and Actinobacteria and with reduced richness (Liu et al., 2017). Late pregnancy stool showed the strongest signs of inflammation and energy loss and when transferred the late pregnancy stool to germ-free mice, it could induce severe inflammation and insulin insensitivity (Koren et al., 2012). Another study involving non-obese and obese individuals found that the obese individuals with a low gut microbiome alpha richness was more correlative with low-grade inflammatory phenotype (Le Chatelier et al., 2013). In our study we found that PE patients demonstrated lower microbial α diversity. We also compared the white blood cell count, percentage of neutrophils and lymphocytes at the time of fecal collection between the two groups and found that there were no obvious statistically differences between the two groups. These studies demonstrated that aberrant gut microbiota maybe related to low-grade inflammation rather than clinical inflammation and indicated that low-grade inflammation was involved in the pathogenesis of PE.

Compared with healthy normal group, microbial beta diversity was significantly different in PE group. At the phylum level, the abundance of Bacteroidetes was significantly lower, while the abundances of Proteobacteria and Fusobacteria were significantly higher in patients with PE. However, some studies showed that the abundance of Bacteroidetes was higher in PE group (Wang et al., 2019; Wang et al., 2020). Furthermore, other researches demonstrated that there was no difference in the abundance of Bacteroidetes between two groups (Lv et al., 2016; Chen et al., 2020). Bacteroidetes is a type of gram-negative bacteria which has been reported to be associated with immunity and metabolic processes (Koliada et al., 2017). The decline in Bacteroidetes relates to a rise in the prevalence of common chronic metabolic disorders, obesity and ulcerative colitis for example (Eckburg et al., 2005; Gomez-Arango et al., 2016). Furthermore, the decrease of abundance was correlated with the severity of the disease (Bohnhoff et al., 1954). However, Gomez-Arango et al. found that Bacteroidetes was negatively correlated with blood pressure and supplied with the bacteria may improve multiple clinical parameters (Saito, 1961).

Our study found that the abundances of Proteobacteria was significantly higher in patients with PE, the result was similar to that of Wang’s results (Funk et al., 2012). The Proteobacteria phylum can lead to a disruption of the gut microbiota in mice and humans (Vollaard et al., 1992; Seksik et al., 2003; Gophna et al., 2006). Similarly, an increased abundance of Proteobacteria is not only observed in humans with severe intestinal inflammation including inflammatory bowel disease, colorectal cancer or necrotizing enterocolitis (Krogius-Kurikka et al., 2009; Morgan et al., 2012; Wang et al., 2012; Normann et al., 2013), but also observed in conditions of low-level intestinal inflammation including irritable bowel syndrome and metabolic syndrome (Bohnhoff et al., 1954; Saito, 1961; Vollaard et al., 1992; Seksik et al., 2003; Eckburg et al., 2005; Gophna et al., 2006; Krogius-Kurikka et al., 2009; Morgan et al., 2012; Wang et al., 2012; Carroll et al., 2012; Le Chatelier et al., 2013; Normann et al., 2013; Lv et al., 2016; Gomez-Arango et al., 2016; Koliada et al., 2017; Wang et al., 2019; Wang et al., 2020; Chen et al., 2020). A previous study performed by Litvak showed that dysbacteriosis of Proteobacteria was a potential diagnostic microbial marker of epithelial dysfunction in the colon (Fei and Zhao, 2013). Our study found that the abundances of Proteobacteria was significantly higher in patients with PE, the result was similar to that of Chen’s results (Funk et al., 2012). The phylum level of Fusobacteria, anaerobic gram-negative rods, are rare agents of severe human diseases and may be associated with colorectal cancer (Litvak et al., 2017). In our study, the relative abundances of Proteobacteria and Fusobacteria were higher in PE group than those in normal groups. The reason may be that these harmful bacteria can lead to disruption of the gut microbiota, release of harmful substances, producing low-level inflammation that involving in PE development.

The F/B ratio was significant higher in the PE group than that in normal group. F/B is considered as a predictive marker of health and diseases (Afra et al., 2013). A system review pointed out that individuals with obesity have a greater F/B ratio, and upon weight loss, this ratio reverts to normal (Mariat et al., 2009; Crovesy et al., 2020). Some previous study also showed that the increased F/B was associated with hypertension, osteoarthritis, and irritable bowel syndrome (Yang et al., 2015; Duan et al., 2019; Chisari et al., 2021). An study thought that hypertension (at least in mice) also involves deficient GPR43 signaling, and insufficient SCFA stilation. The authors found that treatment with the GPCR GPR43 siRNA significantly attenuated the effects of propionate ininhibiting M1 polarization and promoting M2 polarization (Beckers and Sones, 2020).

LEfSe analysis showed that there are 24 differently abundant taxa between the PE group and the healthy group, and these differential taxa may be potential biomarkers. However, they are not specific for PE patients, because they have been demonstrated in other metabolic diseases, and a deeper insight into the microbiome composition may increase the accuracy.

The gut microbiota is significantly different in PE patients, compared with healthy controls in our study. This disruption can lead to the overgrowth of pathogenic infection which in turn trigger an inflammatory immune response including complement and proinflammatory cytokines, leading to the development of PE (Beckers and Sones, 2020). While other studies showed that inflammatory markers were slightly increased in healthy pregnancies and significant increase of inflammatory markers in PE patients suggested that the balance between the inflammatory and anti-inflammatory mechanisms might be disrupted by a shift towards inflammation (Santulli and Al-Mallah, 2019; Mackay and Marques, 2022).

To investigate the role of inflammatory response in PE, we performed analysis of inflammatory factors. TNF-α is a 17‐kDa soluble protein and is secreted primarily not only by monocytes and macrophages but also by a variety of cells, in response to numerous stimuli. It is responsible for the release of proinflammatory cytokines and chemokines, regulating cell apoptosis, and promoting leukocytes activation and proliferation (Guney et al., 2020). Some studies found the similar results. α meta‐analysis demonstrated significantly higher TNF‐α levels in the serum of 1084 pregnant women with PE, compared to 887 with normal pregnancy (Sedger and McDermott, 2014). While another study provided that the mechanism of gut microbiota dysbiosis in PE group was HTR-8/SVneo cellproliferation, invasion, and migration *via* lncRNA BC030099/NF-κB pathway (Xie et al., 2011). High TNF-α levels may be responsible for the anti-vasodilatation effect by inhibiting NO release from endothelium (Xie et al., 2011). The TNF-α inhibitor-etanercept could attenuate elevated blood pressure, supporting the role of TNF-α playing in PE (Guney et al., 2020). IL-6 is a small glycoprotein with effects on inflammation, immune response, and hematopoiesis. Consistent with our study, previous studies demonstrated that IL-6 was significantly higher in PE patients (George and Granger, 2011; Raio et al., 2019; Tang et al., 2022). The decidual cells from PE pregnancies present significantly higher staining for IL-6 compared with placentas derived from normotensive women in an immunohistochemical study, supporting the role of IL-6 in PE (Swellam et al., 2009). Gadonski et al (Jabalie et al., 2019) showed that reduced uterine perfusion pressure rats presented higher arterial pressure and serum IL-6 levels than the normal pregnant controls, supporting the role of IL-6 in elevating blood pressure. TNF-α and IL-6 were found to stimulate the release of sFlt and soluble endoglin (sEng), which are well‐known components of PE pathophysiologic pathways (Žák and Souček, 2019). Other diseases such as infection, pregnancy diabetes (Aggarwal et al., 2019), high-fat diet (Lockwood et al., 2008), obesity (Gadonski et al., 2006) or periodontal disease (Xiang et al., 2022) can cause the increase of serum proinflammatory factor levels. However, our study has excluded patients with acute and chronic infections in the recent period and diabetes during pregnancy. All subjects included in the study were given guidance on diet during pregnancy, and the case group was matched according to pre pregnancy BMI. However, we did not screen the periodontal disease infection of the subjects. In the next step, we will combine the oral flora, intestinal flora and the relationship between preeclampsia.

The limitations of our study should be considered. Firstly, the sample size was relative limited, and a larger sample size are need in further studies. Secondly, this study confirmed that PE patients had significantly different intestinal flora compared with normal pregnancy, but no causal verification experiment was conducted. Thirdly, this study described the gut microbiota dysbiosis in PE patients without further research of the underlying mechanisms. Finally, the effect of dietary differences on gut microbiota outcomes were not considered into this study.

## Conclusion

PE patients demonstrated gut microbiota disturbances and increasing serum proinflammatory factors, providing a new therapeutic strategy for treating PE.

## Data availability statement

The datasets presented in this study can be found in online repositories. The names of the repository/repositories and accession number(s) can be found below: All raw sequences were deposited in the NCBI Sequence Read Archive under accession number SRP394438.

## Ethics statement

The studies involving human participants were reviewed and approved by Ethics Review Board of First Hospital of Hebei Medical University. The patients/participants provided their written informed consent to participate in this study.

## Author contributions

Conception and design of study: YZ, BW, XZ. Acquisition of data: YZ, BW, XZ, DC, SH, HZ. Analysis and/or interpretation of data: YZ, BW, XZ. Drafting and/or revising the manuscript: YZ, BW, XZ. The authors read and approved the final manuscript. All authors contributed to the article and approved the submitted version.
