# Corrigendum: The effect of gut microbiota dysbiosis on patients with preeclampsia

**DOI:** 10.3389/fcimb.2023.1138934

**Published:** 2023-02-09

**Authors:** Yefang Zhao, Bingjie Wang, Xiaoling Zhao, Dan Cui, Shaoke Hou, Hongzhen Zhang

**Affiliations:** ^1^ Department of Obstetrics and Gynecology, First Hospital of Hebei Medical University, Shijiazhuang, Hebei, China; ^2^ Department of Obstetrics, Xingtai People’s Hospital, Affiliated Hospital of Hebei Medical University, Xingtai, Hebei, China

**Keywords:** preeclampsia, gut microbiota, microbial α diversity, microbial β diversity, proinflammatory factor

In the published article, there was an error regarding the affiliation(s) for Yefang Zhao. As well as having affiliation 1, they should also have 2 Department of Obstetrics, Xingtai People’s Hospital, Affiliated Hospital of Hebei Medical University, Xingtai, Hebei, China

The authors apologize for this error and state that this does not change the scientific conclusions of the article in any way. The original article has been updated.

